# MRI-Based Tumor Necrosis Depiction in Pancreatic Ductal Adenocarcinoma: Can It Predict Tumor Aggressiveness?

**DOI:** 10.3390/cancers15082313

**Published:** 2023-04-15

**Authors:** Mark A. Anderson, David E. Knipp, Yoshifumi Noda, Sophia C. Kamran, Vinit Baliyan, Hamed Kordbacheh, Theodore S. Hong, Avinash Kambadakone

**Affiliations:** 1 Department of Radiology, Massachusetts General Hospital, 55 Fruit Street, Boston, MA 02114, USA; 2 Department of Radiology, Gifu University, 1-1-1 Yanagido Street, Gifu City 501-1194, Japan; 3 Department of Radiation Oncology, Massachusetts General Hospital, 55 Fruit Street, Boston, MA 02114, USA

**Keywords:** pancreatic ductal adenocarcinoma, magnetic resonance imaging, necrosis

## Abstract

**Simple Summary:**

Pancreatic adenocarcinoma is a leading cause of cancer related deaths worldwide. Since surgical treatment involves serious risk and morbidity, guiding patients appropriately to surgery for only those that stand to benefit is crucial to avoid unnecessary morbidity. Imaging may provide value in predicting patient prognosis and guiding therapeutic options. Tumor necrosis has been associated with higher tumor grade and worse cancer-specific survival in multiple types of cancers. The purpose of this study was to investigate whether tumor necrosis depicted on abdominal MRI can predict tumor aggressiveness in pancreatic ductal adenocarcinoma. We found that MRI tumor necrosis was associated with higher rates of lymphadenopathy, metastatic disease, and advanced tumor stage compared to pancreatic cancer patients without MRI detectable necrosis. MRI may therefore be prognostically valuable and help guide therapy for patients with pancreatic cancer.

**Abstract:**

The purpose of this study was to investigate whether tumor necrosis depicted on contrast-enhanced abdominal MRI can predict tumor aggressiveness in pancreatic ductal adenocarcinoma (PDAC). In this retrospective analysis, we included 71 patients with pathology-proven PDAC who underwent contrast-enhanced MRI from 2006 to 2020. Assessment for the presence/absence of imaging detected necrosis was performed on T2-weighted and contrast-enhanced T1-weighted images. Primary tumor characteristics, regional lymphadenopathy, metastases, stage, and overall survival were analyzed. Fisher’s exact and Mann-Whitney *U* tests were used for statistical analysis. Of the 72 primary tumors, necrosis was identified on MRI in 58.3% (42/72). Necrotic PDACs were larger (44.6 vs. 34.5 mm, *p* = 0.0016), had higher rates of regional lymphadenopathy (69.0% vs. 26.7%, *p* = 0.0007), and more frequent metastases (78.6% vs. 40.0%, *p* = 0.0010) than those without MRI-evident necrosis. A non-statistically significant reduction in median overall survival was observed in patients with versus without MRI-evident necrosis (15.8 vs. 38.0 months, *p* = 0.23). PDAC tumor necrosis depicted on MRI was associated with larger tumors and higher frequency of regional lymphadenopathy and metastases.

## 1. Introduction

Pancreatic ductal adenocarcinoma (PDAC) is the twelfth most common cancer worldwide, the fourth leading cause of cancer related deaths in the United States, and accounts for 94% of all primary pancreatic cancers [1]. PDAC carries a high mortality rate, with a reported 5-year survival of 8% [2]. The only curative treatment remains surgical resection; however, as many patients present late, only 20% are surgical candidates [3]. Imaging, particularly cross-sectional imaging techniques such as computed tomography (CT) and magnetic resonance imaging (MRI), play a critical role in the diagnosis, treatment planning, and monitoring treatment response in patients with PDAC. 

Several authors have investigated the role of tumor margin, size, vascular invasion, and regional lymphadenopathy as depicted on imaging with tumor grade and overall prognosis [4,5,6]. However, whether internal tumor morphology has value in determining tumor grade and patient outcome is not well-established. One such morphological feature for PDAC is tumor necrosis. Tumor necrosis has already been reported to be associated with higher tumor grade and worse cancer-specific survival in renal, colorectal, lung, and breast cancers [7,8,9,10].

Rim-enhancing PDAC on dynamic contrast-enhanced abdominal MRI and poorly enhanced areas in PDAC on contrast-enhanced abdominal CT have been associated with significantly worse disease-free survival (DFS) and overall survival (OS) [11,12]. Rim-enhancing PDACs with intratumoral fluid signal also have been associated with more aggressive histologic tumor grades, less frequent remaining normal parenchymal acini, and more frequent pathologic tumor necrosis in patients who underwent surgery [13,14]. When imaging necrosis is defined as an intratumoral central fluid signal intensity region on T2-weighted MR images with irregular, peripheral enhancement on dynamic-enhanced MR images, patients with necrotic PDACs treated with upfront surgery have more frequent histological necrosis, more aggressive tumor differentiation, and significantly shorter DFS and OS than those without MRI detected necrosis [14]. However, surgical candidates only represent a minority of patients with PDAC. The purpose of this study was to investigate if tumor necrosis identified on contrast-enhanced abdominal MRI can predict tumor aggressiveness in both resectable and unresectable PDACs.

## 2. Materials and Methods

This investigation was approved by the Institutional Review Board and was compliant with the guidelines of the Health Insurance Portability and Accountability Act (HIPAA). A retrospective search of an institutional radiology database from December 2006 to March 2020 was performed to identify patients with pathologically confirmed PDAC who also underwent contrast-enhanced MRI. Patients were excluded if MRI was obtained after chemotherapy; if the PDAC was not visible or included in the field of view on MRI; if poor image quality or artifact precluded PDAC evaluation; or if PDAC arose within an intraductal papillary mucinous neoplasm. 

All patients underwent a contrast-enhanced MRI on a 1.5 Tesla (n = 53) or 3 Tesla (n = 18) magnet. Six exams were initially performed at an outside institution and uploaded to our picture archival and communication system (PACS). Institutional protocol of the upper abdomen included: pre- and multiphasic post-contrast fat-suppressed T1-weighted imaging (repetition time (TR) 3.4 ms, echo time (TE) 1.4 ms, inversion time (TI) 5–13 ms, flip angle 10–25°, matrix size 160 × 160, field of view (FOV) 320–480 mm, slice thickness 2–5 mm with 2–3 mm gap, and number of excitations (NEX) 0.7–1); fat-suppressed, respiratory-triggered spin-echo T2-weighted imaging (TR infinite, TE 84.6 ms, flip angle 90–160°, matrix size 256 × 128, FOV 320–440 mm, slice thickness 4–8 mm with 4–10 mm gap, and NEX 1–2); and diffusion-weighted imaging (DWI) (TR 3,000 ms, TE 74.6 ms, flip angle 90°, matrix size 128 × 128, FOV 320–420 mm, slice thickness 5–8 mm with 5–10 mm gap, and NEX 1–2). Contrast volume and agents included: 20 mL gadopentetic acid (Bayer, Germany) (n = 52), 10 mL gadoxetic acid (Bayer) (n = 11), 20 mL gadobenic acid (Bracco Diagnostics Inc., New Jersey) (n = 2), 20 mL gadobutrol (Bayer) (n = 1), and non-recorded (n = 5). Post-contrast images were obtained in the arterial (25–35 s), portal venous (70 s), and equilibrium/late (180 s) phases. Hepatobiliary phase imaging was performed at 20 min following injection in 11 patients who received gadoxetic acid. 

**Figure 1:**
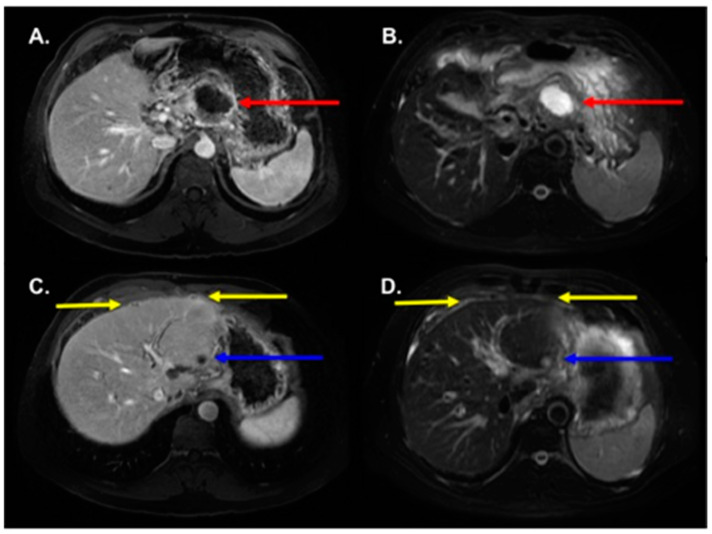
A 48-year-old male with a necrotic mass in the pancreatic body. (**A**) Axial T1 fat-suppressed portal venous phase image demonstrates central hypo-enhancement (red arrow). (**B**) Axial T2 fat-suppressed image shows associated hyperintensity (red arrow). (**C**,**D**) Hypo-enhancing, T2 hyperintense lesions are seen within the liver (blue arrows) and along the anterior hepatic capsule (yellow arrows). Endoscopic ultrasound biopsy revealed primary PDAC with necrosis.

**Figure 2:**
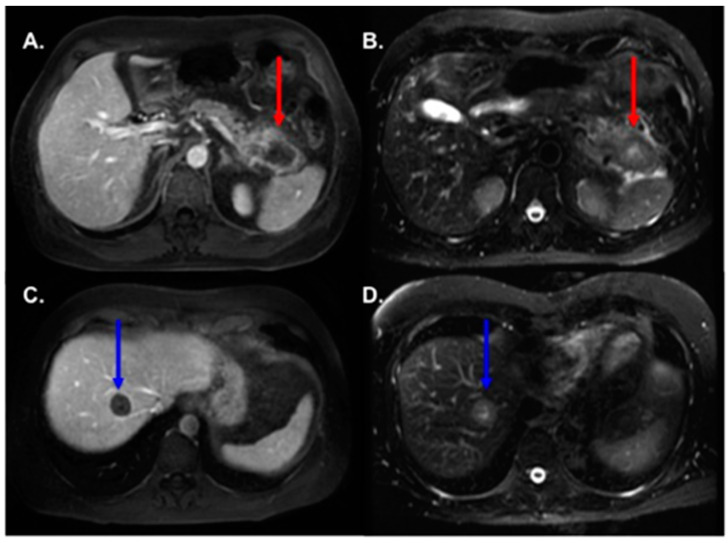
A 51-year-old male with a necrotic mass in the tail of the pancreas. (**A**) Axial T1 fat-suppressed portal venous phase image demonstrates central hypo-enhancement (red arrow). (**B**) Axial T2 fat-suppressed image shows associated hyperintensity (red arrow). (**C**,**D**) A hypo-enhancing, T2 hyperintense lesion is seen within the liver (blue arrows). Endoscopic ultrasound biopsy revealed primary PDAC with necrosis.

Image analysis was performed by a board-certified abdominal radiologist with 11 years of experience (blinded) and an abdominal fellowship trained radiologist (blinded) in consensus on a PACS workstation. A predesigned template was used for image analysis. The following tumor metrics were recorded: tumor location; tumor margin (well-defined, partially well-defined, or ill-defined); tumor size at largest long-axis dimension on axial images; tumor stage; intrinsic T1 and T2 signal intensity (hypointense, isointense, or hyperintense relative to normal background pancreatic parenchyma); enhancement characteristics (hypo-enhancement, iso-enhancement, or hyper-enhancement compared with normal pancreas); presence or absence of pancreatic and biliary ductal dilatation; pancreatic parenchymal atrophy upstream from the tumor; extra-pancreatic tumor extension; vascular involvement; loco-regional lymphadenopathy (defined as > 1 cm short-axis); and the presence of definitive metastatic disease (liver, peritoneum and distant organs). The evaluation of vascular involvement followed guidelines set forth by the American Joint Commission on Cancer (AJCC) staging criteria of PDAC, eighth edition and National Comprehensive Cancer Network, Version 2.2017 [15]. Unresectable arterial encasement was defined as encasement or greater than 180° contact with the celiac axis (CA) or superior mesenteric artery (SMA). Venous invasion was defined as unreconstructable superior mesenteric vein (SMV) or portal vein (PV) due to extensive encasement/occlusion. In those patients with DWI available, apparent diffusion coefficient (ADC) value was measured. A normalized ADC value was obtained on the highest provided *b*-value dataset (range: 500–800 s/mm²) by calculating the ratio of values from the primary tumor and a region of interest (ROI) within the spleen, with careful attention to avoid vessels or focal splenic pathology. The observers recorded the presence or absence of tumor necrosis, defined as portions of pancreatic tumors demonstrating areas of T1 hypointensity/T2 hyperintensity and lack of enhancement on multiphasic images (Figure 1 and Figure 2) [16,17]. The size of tumor necrosis was recorded, and the area of tumor involved by necrosis was estimated. 

An independent observer reviewed the medical records for each patient including oncology notes, surgical reports, radiology reports, and pathology results. Patient demographics, date and method of tissue sampling, and tumor staging were recorded. Additionally, radiological exams performed within 30 days of the index MRI were also reviewed to evaluate for the presence of distant metastases. 

All statistical analysis was performed using the MedCalc version 19.2 software program for Windows (MedCalc Software, Ostend, Belgium). Mann-Whitney *U* and Fisher’s exact tests were used to compare patient background factors and tumor characteristics including patients’ age, sex, tumor location, tumor stage, and the presence of metastatic disease, and additionally, MRI findings including: tumor margins; tumor size; T1 and T2 signal intensity; enhancement characteristics at each phase; pancreatic and biliary ductal dilatation; pancreatic tail atrophy; extra-pancreatic extension; vascular involvement; and loco-regional lymphadenopathy between the necrotic and non-necrotic tumors. Mann-Whitney *U* test was conducted to compare ADC values between the necrotic and non-necrotic tumors. The Kaplan–Meier method and log-rank test were performed to compare the OS and between patients with necrotic and non-necrotic tumors. A *p*-value of ≤ 0.05 was considered statistically significant.

## 3. Results

### 3.1. Patients’ Demographics and Tumor Characteristics

In total, 101 patients were pathologically confirmed as having PDAC and subsequent abdominal MRI. Overall, 30 of 101 patients were excluded for 1 of the following reasons: MRI was obtained after chemotherapy (n = 27), PDAC was not visible on MR images (n = 1), poor image quality to the degree that precluded tumor evaluation (n = 1), and PDAC arising in an intraductal papillary neoplasm (n = 1). After the exclusion of these patients, the final cohort included 71 patients (mean age, 63.3 ± 10.0 years; age range, 43–85 years) with 72 pathologically proven PDACs (1 patient had 2 synchronous PDACs at presentation). Of these, 47 were male (mean age, 62.1 ± 10.2 years; age range, 43–84 years) and 24 were female (mean age, 65.7 ± 9.5 years; age range, 51–85 years).

Patient background factors and tumor characteristics are summarized in Table 1.

Tissue sampling was performed an average of 17.7 ± 28.4 days from time of MRI, and was obtained via endoscopic ultrasound (78%, n = 56/72), surgical resection (13%, n = 9/72), or percutaneous core biopsy (9%, n = 7/72). In 6 of the percutaneously biopsied tumors, pathology was obtained from a liver metastasis (n = 5), or a peritoneal metastasis (n = 1). In 12 tumors that were diagnosed by endoscopic ultrasound (n = 10) or surgical resection (n = 2), pathological confirmation was available for determination of histological primary tumor necrosis. 

Intra-tumoral necrosis was identified on MRI in 58.3% (42/72) of tumors, with an average 57.8% necrotic component as defined by the longest axial dimension. Tumor location was significantly different between the necrotic and non-necrotic tumors (*p* = 0.010). Overall, 1% (1/72) of tumors were categorized as Stage IA, 10% (7/72) as Stage IB, 1% (1/72) as Stage IIA, 8% (6/72) as Stage IIB, 7% (5/72) as Stage III, and 73% (52/72) as Stage IV. Necrotic tumors were associated with more advanced stage than non-necrotic tumors (*p* = 0.023). Metastatic disease was seen in 63% (n = 45/72) of patients, most commonly involving the liver (n = 42), lung (n = 7), peritoneum (n = 6), adrenal gland (n = 2), and bone (n = 1). Necrotic tumors had a significantly higher rate of having distant metastases than non-necrotic tumors (*p* = 0.0001). No significant difference was found in patients’ age (*p* = 0.49) and sex (*p* = 0.62) between necrotic and non-necrotic tumors.

### 3.2. MRI Findings

MRI findings of necrotic and non-necrotic tumors are summarized in Table 2. 

**Figure 3:**
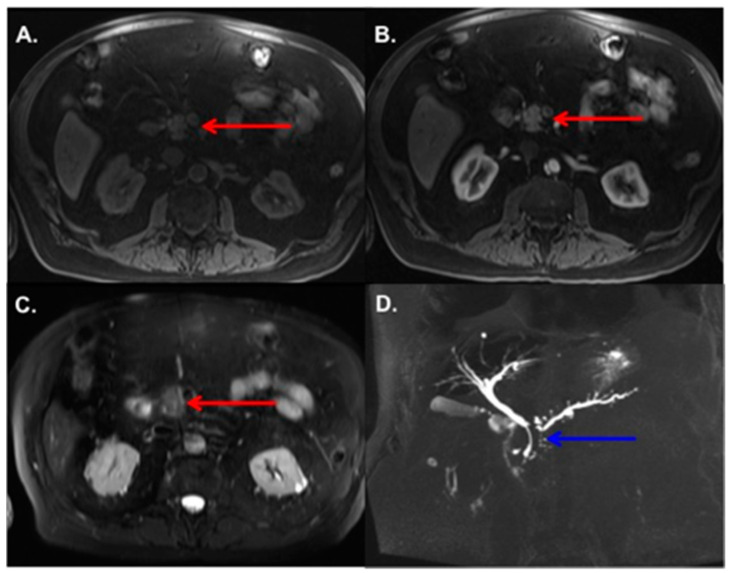
A 82-year-old male with a non-necrotic PDAC in the pancreatic head. (**A**) Axial T1 fat-suppressed image demonstrates hypointensity (red arrow). (**B**) Arterial phase image shows hypo-enhancement (red arrow). (**C**) T2 fat-suppressed image shows isointense signal (red arrow). (**D**) MRCP demonstrates narrowing of the lower common bile duct and pancreatic duct with upstream dilatation (blue arrow). No metastatic disease or lymphadenopathy was identified. The tumor was resected and no necrosis found.

Tumor size was significantly greater in necrotic tumors than in non-necrotic tumors (*p* = 0.0016). Necrotic tumors had a significantly higher rate of loco-regional lymphadenopathy than non-necrotic tumors (*p* = 0.0007). Pancreatic parenchymal atrophy upstream from the tumor (*p* = 0.031) and ill-defined margins (*p* = 0.030) were more frequent in non-necrotic tumors than in necrotic tumors. No significant difference was seen with regards to T1 signal intensity (*p* = 0.084), arterial enhancement characteristic (*p* = 0.42), presence of pancreatic (*p* = 0.13) and biliary (*p* = 0.61) ductal dilatation, extra-pancreatic extension (*p* = 0.37), vascular involvement (*p* = 0.23), and ADC values (*p* = 0.28) between necrotic and non-necrotic tumors (Figure 3). 

### 3.3. Radiologic–Pathologic Correlation

Among the 12 tumors that could be pathologically evaluated for tumor necrosis, necrosis was identified in 10 tumors. MRI could detect tumor necrosis in 80% (n = 8/10). In two tumors not identified to have necrosis on pathology, one was false positive for necrosis by MRI. There was no mention of tumor necrosis in 83% tumors (n = 60); however, this is not routinely commented upon with every sample as standard of care at our institution. 

### 3.4. Patients Overall Survival

The median patient OS was 20.6 months. Moreover, 1-year, 2-year, and 3-year OS rates were 67%, 50%, and 39%, respectively. Median OS was lower in patients with necrotic tumors (median OS, 16.8 months) than those with non-necrotic tumors (median OS, 38.0 months); however, there was no statistical significance in OS between patients with necrotic and non-necrotic tumors (hazard ratio, 1.57; 95% confidence interval, 0.75–3.28) (*p* = 0.23).

## 4. Discussion

Tumor necrosis serves as an independent marker for local aggressiveness and overall worse prognosis in a variety of solid tumors including renal, colorectal, lung, and breast [7,8,9,10]. In the current investigation, tumor necrosis detected by MRI in patients with PDAC was associated with significantly higher rates of loco-regional lymphadenopathy, metastatic disease, and advanced tumor stage compared to PDAC without MRI detectable necrosis. 

Previous pathological studies [18] have shown that pancreatic tumor necrosis on post-operative histologic assessment correlates with positive nodal status and the presence of metastatic disease, as well as serves as an independent predictor of shorter DFS. We also found that the presence or absence of tumor necrosis in PDAC as detected by pre-operative MRI was significantly more frequently accompanied by loco-regional lymphadenopathy, metastatic disease, and advanced stage compared to tumors without MRI detected necrosis. This suggests that pre-operative MRI can provide meaningful prognostic value that correlates with post-operative histologic data.

It is hypothesized that tumor necrosis results from cellular hypoxia mediated signaling, such as hypoxia-inducible factor-1α (HIF-1α) and the expression of carbonic anhydrase IX (CAIX), which results in tumor dedifferentiation, angiogenesis, and a more aggressive histological phenotype [18,19]. A previous study demonstrated that greater tumor size and rim-enhancement, thought to represent tumor necrosis, are associated with lower DFS and OS in patients with operable PDAC [13]. In our study, although no statistically significant difference was found in OS between patients with necrotic and non-necrotic tumors, median OS in patients with non-necrotic tumors was longer than in those with necrotic tumors (38.0 months vs. 16.8 months). Overall, 72% (52/72) of tumors were categorized in stage IV in the present study. It may be that OS was not significantly different between patients with necrotic and non-necrotic tumors because most patients had more advanced PDAC compared to the previous study. Additionally, an increase in median OS of more than 20 months in patients with advanced stage PDAC may be meaningful despite lack of statistical significance.

Several prior studies have shown that cellular hypoxia is associated with reduced radiosensitivity and resistance to chemotherapy [20,21]. Identifying primary tumor necrosis in PDAC on MRI may therefore have important consequences for both treatment planning and prognosis. If primary tumor necrosis is found on pre-treatment MRI, a high index of suspicion for presence of locoregional lymphadenopathy and metastatic disease should be maintained by the interpreting radiologist. Even if metastases are not initially found by imaging, their strong association with necrosis and shorter OS may make pursing initial non-invasive therapy, such as with neoadjuvant chemoradiation therapy, a more reasonable option. Development of predictive biomarkers that can help guide therapeutic strategies can help avoid unnecessary morbidity in patients with PDAC [22].

This study has limitations. First, because many of our samples were obtained via EUS or percutaneous biopsy, gross pathology was not always available to confirm the presence of necrosis suspected on MRI. Of the surgical specimens obtained, necrosis was also not a routinely reported pathologic feature. However, the reliability of contrast enhanced MRI in the evaluation of tumor necrosis has been widely reported in the literature. As in this study, the lack of enhancement and presence of central T2 hyperintensity are two commonly used markers [16,23,24]. Second, though we excluded one case of PDAC arising in intraductal papillary neoplasm, we could not completely differentiate tumor necrosis from cystic or mucinous components in patients who did not have surgical specimens. A larger study with complete pathological correlation would be of value to validate our results. Lastly, we only assessed the burden of lymphadenopathy and metastatic disease at the time of presentation. Analysis of subsequent disease burden is beyond the scope of this study, as we focused on preoperative information and prognostic value.

## 5. Conclusions

In conclusion, primary tumor necrosis seen on contrast-enhanced MRI at time of presentation is associated with a significant increase in frequency of regional lymphadenopathy, metastatic disease, and consequently, advanced stage of disease in patients with resectable and unresectable PDAC.

## Figures and Tables

**Table 1:** Comparison of patient and tumor characteristics between necrotic and non-necrotic pancreatic ductal adenocarcinomas.

Feature	Necrotic (n = 42)	Non-Necrotic (n = 30)	*p* Value
Age (Years)	62.8 ± 10.1 (47–85)	64.3 ± 10.0 (43–83)	0.49
Sex (Male: Female)	26:15	21:9	0.62
Location			0.01 *
Head	16 (38%)	15 (50%)	
Uncinate	8 (19%)	1 (3%)	
Neck	5 (12%)	1 (3%)	
Body	6 (14%)	12 (40%)	
Tail	7 (17%)	1 (3%)	
Tumor Stage			0.02 *
IA	0 (0%)	1 (3%)	
IB	4 (10%)	3 (10%)	
IIA	1 (2%)	0 (0%)	
IIB	1 (2%)	5 (17%)	
III	1 (2%)	4 (13%)	
IV	35 (84%)	17 (57%)	
Metastases	33 (79%)	12 (40%)	0.0001 *
Liver	32	10	
Lung	5	2	
Peritoneum	5	1	
Adrenal	1	1	
Bone	1	0	

Data are means ± 1 standard deviation with ranges in parentheses. * *p* ≤ 0.05, significant difference.

**Table 2:** Comparison of MRI findings between necrotic and non-necrotic PDAC.

Feature	Necrotic (n = 42)	Non-Necrotic (n = 30)	*p* Value
Margins			0.03 *
Ill-Defined	16 (38%)	21 (70%)	
Partially Defined	22 (52%)	8 (27%)	
Well-Defined	4 (10%)	1 (3%)	
Tumor Size (mm)	44.6 ± 15.7 (21.7–96.8)	34.5 ± 19.6 (14.0–82.0)	0.002 *
T1 Signal Intensity			**0.08**
Hypointense	36 (86%)	20 (67%)	
Isointense	6 (14%)	10 (33%)	
T2 Signal Intensity			<0.0001 *
Hypointense	3	13	
Isointense	7	15	
Hyperintense	32	2	
Arterial Enhancement			0.42
Hypo-enhancement	42 (100%)	29 (97%)	
Iso-enhancement	0 (0%)	1 (3%)	
PV Enhancement			0.0005 *
Hypo-enhancement	42 (100%)	22 (73%)	
Iso-enhancement	0 (0%)	8 (27%)	
Equilibrium Enhancement			<0.0001 *
Hypo-enhancement	42 (100%)	15 (50%)	
Iso-enhancement	0 (0%)	15 (50%)	
PD Dilatation	31 (74%)	27 (90%)	0.13
CBD Dilatation	12 (29%)	11 (37%)	0.61
Pancreatic Tail Atrophy	16 (38%)	20 (67%)	0.03 *
Extra-Pancreatic Extension	32 (76%)	26 (89%)	0.37
Vascular Involvement	24 (57%)	12 (40%)	0.23
Lymphadenopathy	29 (69%)	8 (27%)	0.0007 *
ADC value (×10^−3^ mm^2^/s)	1.51 ± 0.58 (0.37–3.45)	1.48 ± 0.72 (0.37–3.63)	0.28

PV: portal venous. PD: pancreatic duct. CBD: common bile duct. ADC: apparent diffusion coefficient. Data are means ± 1 standard deviation with ranges in parentheses. * *p* ≤ 0.05, significant difference.

## Data Availability

The data presented in this study may be available on request from the corresponding author. The data are not publicly available due to data patient privacy concerns.
